# Vitamin D Receptor Gene Polymorphisms Are Associated with Obesity and Inflammosome Activity

**DOI:** 10.1371/journal.pone.0102141

**Published:** 2014-07-14

**Authors:** Nasser M. Al-Daghri, Franca R. Guerini, Omar S. Al-Attas, Majed S. Alokail, Khalid M. Alkharfy, Hossam M. Draz, Cristina Agliardi, Andrea S. Costa, Irma Saulle, Abdul Khader Mohammed, Mara Biasin, Mario Clerici

**Affiliations:** 1 Biomarkers Research Program, Biochemistry Department, College of Science, King Saud University, Riyadh, Kingdom of Saudi Arabia (KSA); 2 Prince Mutaib Chair for Biomarkers of Osteoporosis, College of Science, King Saud University, Riyadh, KSA; 3 Center of Excellence in Biotechnology Research, King Saud University, Riyadh, KSA; 4 Don Gnocchi Foundation, ONLUS, Milano and Università degli Studi di Milano, Milano, Italy; 5 Clinical Pharmacy Department, College of Pharmacy, King Saud University, Riyadh, KSA; 6 INRS-Institute Armand Frappier, University of Quebec, Laval, Quebec, Canada; 7 Department of Biomedical and Clinical Sciences, Università degli Studi di Milano, Milano, Italy; Sanjay Gandhi Medical Institute, India

## Abstract

To explore the mechanisms underlying the suggested role of the vitamin D/vitamin D receptor (VDR) complex in the pathogenesis of obesity we performed genetic and immunologic analyses in obese and non-obese Saudi individuals without other concomitant chronic diseases. Genomic DNA was genotyped for gene single nucleotide polymorphisms (SNPs) of VDR by allelic discrimination in 402 obese (body mass index –BMI≥30 kg/m2) and 489 non-obese (BMI<30 kg/m2) Saudis. Q-PCR analyses were performed using an ABI Prism 7000 Sequence Detection System. The inflammosome pathway was analysed by PCR, cytokines and plasma lipopolysaccaride (LPS) concentrations with ELISA assays. Results showed that the VDR SNPs rs731236 (G) (TaqI) and rs1544410 (T) (Bsm-I) minor allele polymorphisms are significantly more frequent in obese individuals (p = 0.009, β = 0.086 and p = 0.028, β = 0.072, respectively). VDR haplotypes identified are positively (GTA) (p = 0.008, β = 1.560); or negatively (ACC) (p = 0.044, β = 0.766) associated with obesity and higher BMI scores. The GTA "risk" haplotype was characterized by an up-regulation of inflammosome components, a higher production of proinflammatory cytokines (p<0.05) and a lower VDR expression. Plasma LPS concentration was also increased in GTA obese individuals (p<0.05), suggesting an alteration of gut permeability leading to microbial translocation. Data herein indicate that polymorphisms affecting the vitamin D/VDR axis play a role in obesity that is associated with an ongoing degree of inflammation, possibly resulting from alterations of gut permeability and microbial translocation. These results could help the definition of VDR fingerprints that predict an increased risk of developing obesity and might contribute to the identification of novel therapeutic strategies for this metabolic condition.

## Introduction

Obesity is a complex disease that affects over 500 million people worldwide and whose incidence increased substantially in the past 3 decades [1]. The prevalence of this disease differs in diverse zones of the world and peaks in countries such as Saudi Arabia, where 34% of adults are obese [2]. Obesity is associated with premature death due to an increased risk of a number of chronic conditions including type 2 Diabetes Mellitus (T2DM) and cardiovascular disease [3]–[4], and is correlated with an ongoing degree of low grade inflammation [5].

The pathogenesis of this disease is complex and includes genetic and environmental factors that are not yet fully clarified. Vitamin D is a good candidate to play a role in obesity as evidenced by epidemiologic, genetic and metabolic data [6]–[8]. The action of this vitamin is mediated through vitamin D receptor (VDR), a nuclear transcription-regulating factor that drives the synthesis of proteins involved in bone mineral homeostasis and cell cycle regulation. Vitamin D is a seco-steroid hormone ingested in the diet or synthesized in the skin when 7-dehydrocolesterol reacts with UVB ultraviolet light. The biologically inert vitamin D_3_ is hydroxylated in the liver and in the kidney into (1,25(OH)_2_D). This form becomes active upon binding to the vitamin D receptor (VDR). Thus, given the metabolic pathway of vitamin D synthesis and action, this compound can be described as an environmental factor (diet, UVB light) whose metabolic effects are influenced by the genetic background (genetic variance of VDR). One of the main evidence for a role of VDR in obesity was derived from transgenic mice studies that over-express human VDR in adipocytes which leads to a marked decreases in energy expenditure and induction of obesity [9], [10].

Besides being involved in the pathogenesis of obesity, the vitamin D/VDR axis also modulates inflammation. In vitro studies showed that VDR agonists decrease pro- inflammatory cytokine production by human blood mononuclear cells [11], and that pretreatment of rat peritoneal cells with 1,25 (OH)_2_D_3_ inhibits high glucose- and lipopolysaccaride (LPS)-induced tumor necrosis factor alpha (TNFα) as well as tumor growth factor beta (TGFβ) release [12]. Recent results indicate that the down regulation of VDR expression by siRNA leads to an increased production of pro-inflammatory cytokines by human adipocytes [13]. These effects are possibly mediated by the modulation of the inflammosome, a multiprotein complex expressed in myeloid cells that is part of the innate immune response. The inflammosome includes a cascade of proteins whose activation results in the production of inflammatory cytokines including interleukin 1β (IL1β) and IL18. Even acute inflammation in adipose tissues was suggested to lead to changes in the number and the quality of stromal cells, a phenomenon known as "adipose tissue remodeling" [14].

The VDR gene contains several polymorphisms, including three single nucleotide polymorphisms (SNPs) located near the 3′ un-translated region (3′ UTR) (*Taq1*, *Bsm1*, and *Apa1*) identified by their restriction endonuclease sites [15]. Although not functional, these SNPs are linked with a poly (A) microsatellite repeat in the 3′ UTR that could influence VDR mRNA stability [16]. Given the metabolic pathway of vitamin D synthesis and action, this compound can be described as an environmental factor whose metabolic effects are influenced genetically.

We investigated possible associations of the three known VDR SNPs rs731236(A/G)(TaqI), rs1544410(C/T)(BsmI) and rs7975232 (A/C)(ApaI) with obesity in a population from Saudi Arabia, a country where obesity is reaching endemic proportions [2]. As VDR modulates inflammation, which in turn is observed in obesity, we verified whether the VDR SNPs that are more common in obese individuals would be associated with an up-regulation of inflammosome-related proteins and cytokines. Results herein reinforce the concept that the vitamin D/VDR axis plays a role in obesity which is at least partially mediated by an ongoing degree of inflammation.

## Results

### VDR polymorphisms in obese individuals

Analyses of possible association of VDR minor allele polymorphisms with BMI were performed in 891 Saudi subjects (402 obese, 489 lean) through a linear regression analysis considering BMI as the independent variable, and gender and age as covariates. The association of these variables with obesity and BMI was indicated by β positive values. Variables (including age and VDR minor alleles) with a positive β value are associated with higher BMI, therefore to a higher risk of obesity after adjustment for all the other covariates. Conversely, β values less than zero were associated with lower BMI scores and identify individuals with a lower risk to develop obesity (in case they are not obese) or are overweight. Results of these analyses showed that the VDR rs731236 (G) and VDR rs1544410 (T) alleles were positively associated with BMI and these alleles were associated with obesity independent of age and sex (*p* = 0.009, β = 0.086 and *p* = 0.028, β = 0.072 respectively) (Table 1).

**Table 1:** VDR polymorphisms association with BMI scores.

		t	p value	β	95% CI
Model 1		23.39	<0.001	27.87	21.75–25.05
	Age	**6.46**	**<0.001**	**0.212**	**0.08–0.15**
	Gender	**−2.26**	**0.024**	**−0.074**	**−2.05–−0.15**
	rs731236(G)	**2.625**	**0.009**	**0.086**	0.33–2.29
Model 2		28.35	<0.001	23.59	21.95–25.22
	Age	**6.41**	**<0.001**	**0.211**	**−0.08–−0.15**
	Gender	**−2.25**	**0.025**	**−0.074**	**−2.04–−0.14**
	rs1544410(T)	**2.20**	**0.028**	**0.072**	**0.12–2.06**
Model 3		24.93	<0.001	23.41	21.56–25.25
Selected Variables	Age	**6.36**	**<0.001**	**0.21**	**0.08–0.15**
	Gender	**−2.26**	**0.024**	**−0.074**	**−2.05–−1.14**
	rs7975232(A)	1.67	0.095	0.055	−0.18–2.30

Linear regression analyses were used to compare the VDR genotypes adjusted for the co-variants age and gender. Significance was set at p<0.05. Responsible variable: BMI scores, covariates: age and gender (female vs male). Model 1: VDR rs731236(G):AG/GG *vs.* AA; Model 2: VDR rs1544410(T): CT/TT *vs.* CC; Model 3: VDR rs7975232(A):AC/AA *vs*. CC.

Haplotype association with obesity was evaluated by verifying the distribution of VDR haplotypes in obese *vs.* lean subjects. Results showed that the VDR rs731236 (G)/rs1544410 (T)/rs7975232 (A)(GTA) haplotype was more frequent in obese individuals, whereas the complementary rs731236 (A)/rs1544410 (C)/rs7975232(C)(ACC) haplotype was more common in lean subjects (Table 2).

**Table 2:** VDR Haplotype distribution. Percentage frequencies are reported; VDR Haplotype association with linear BMI accounting for age and gender

Haplotype	Obese%	Lean%	P-value	Linear BMI association		
				β	Stat	P-value
ATC	0.6	0.2	-	−0.873	1.63	0.202
ACC	36.0	38.0	0.227	−0.766	3.90	0.044
GTA	40.4	36.3	0.075	1.56	7.09	**0.008**
ATA	1.5	2.4	-	0.172	0.04	0.831
GCA	2.6	3.6	0.604	0.352	0.776	0.379
ACA	18.7	19.3	0.677	−0.438	0.73	0.393

Order of SNPs: rs731236, rs1544410, rs7975232. Pearson‘s p value, beta risk and statistic were calculated by Haplotype analysis.

Haplotype stratification by BMI values was calculated next by linear regression analyses in the total group of subjects adjusted for age and sex. A statistically significant association was detected between the VDR GTA haplotype and positive β values (*p* = 0.008, β = 1.560). This haplotype is thus associated with a higher risk of obesity and higher BMI scores. Notably, the complementary ACC haplotype was negatively associated with BMI (*p* = 0.044, β = −0.766) (Table 2). The ACC haplotype, therefore, was significantly associated with lack of obesity in non-obese individuals and with lower BMI scores in obese subjects.

### Inflammosome pathway expression

**Figure 1:**
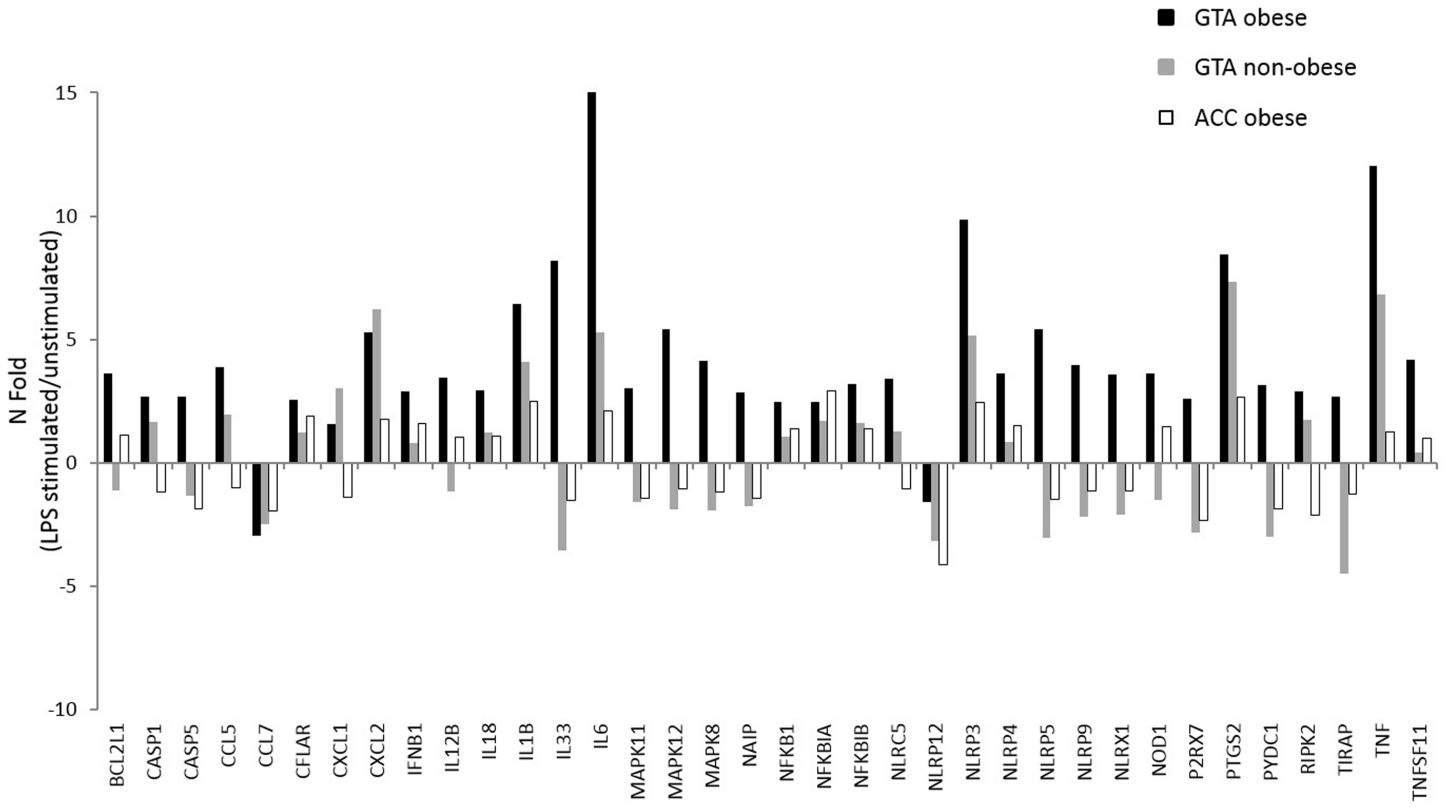
Expression of 96 genes involved in the inflammosome pathway assessed by real-time quantitative RT-PCR in obese and non-obese subjects carrying the VDR “at-risk” (GTA) or the “protective” (ACC) haplotype. Following LPS stimulation the expression of genes within the inflammosome pathway is significantly more increased in obese individuals with the GTA “risk” haplotype. Results are shown as fold-change expression compared to the ACC non obese controls assumed as baseline expression level. Only the targets showing different expression levels in the different groups are shown.

The inflammosome pathway was evaluated in cells of obese and non-obese individuals who were characterized by the presence of the “protective” (ACC) or the “risk” (GTA) VDR haplotypes; cells were stimulated with LPS. Results obtained in non-obese individuals carrying the ACC VDR haplotype were used as the baseline value. An increased expression of genes within the inflammosome pathway was observed in GTA non-obese as well as in both ACC and GTA obese individuals, but the up regulation was significantly more marked in obese individuals with the GTA “risk” haplotype (Figure 1). Within this VDR haplotype, mRNA expression for a number of genes, including the inflammosome components (NAIP, CASP5, NLRP3), Nod-like receptors (NAIP, NLRX1, NOD1, NLRC5 NLRP3, NLRP4, NLRP5 NLRP9), downstream signaling (P2RX7, PANX1, RIPk2, TIRAP, NFKB1) and effector molecules (IL12β, IL18, IL1β, IL33, PTGS2 CCL5, CCL7, IL6, TNF, IFNβ) was increased in obese individuals carrying the GTA “risk” haplotype.

### Pro-inflammatory cytokine production

**Figure 2:**
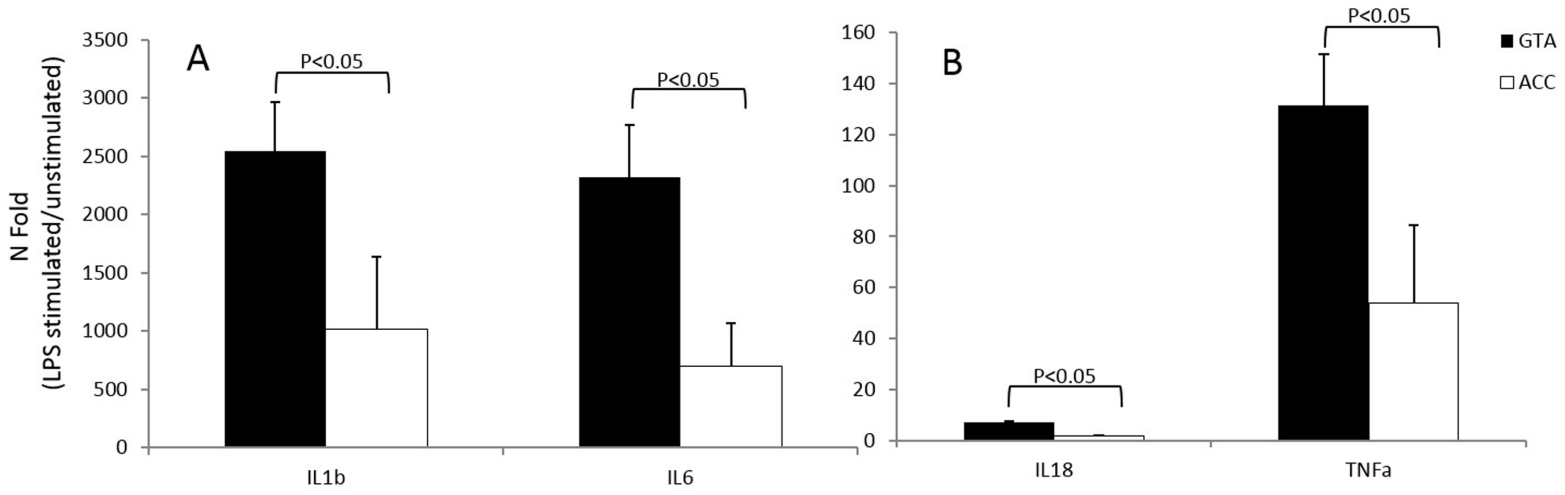
IL1β, IL6 (panel A) IL18 and TNFα (panel B) production assessed by multiplex ELISA in supernatant of LPS stimulated PBMC isolated from obese individuals carrying the VDR “at-risk” (GTA)(black bars) or the “protective” (ACC)(white bars) haplotype. Results are shown as mean values fold-change expression from the unstimulated sample ± standard error.

Activation of the inflammosome results in the down-stream production of a number of proinflammatory cytokines. The higher inflammatory response observed in individuals carrying the GTA “risk” VDR haplotype was further confirmed by ELISA analyses showing that IL1β, IL6, TNFα and IL18 production by LPS-stimulated PBMC was significantly increased (*p*<0.05) by cells of obese individuals carrying this haplotype (Figure 2).

### Plasma LPS concentration

**Figure 3:**
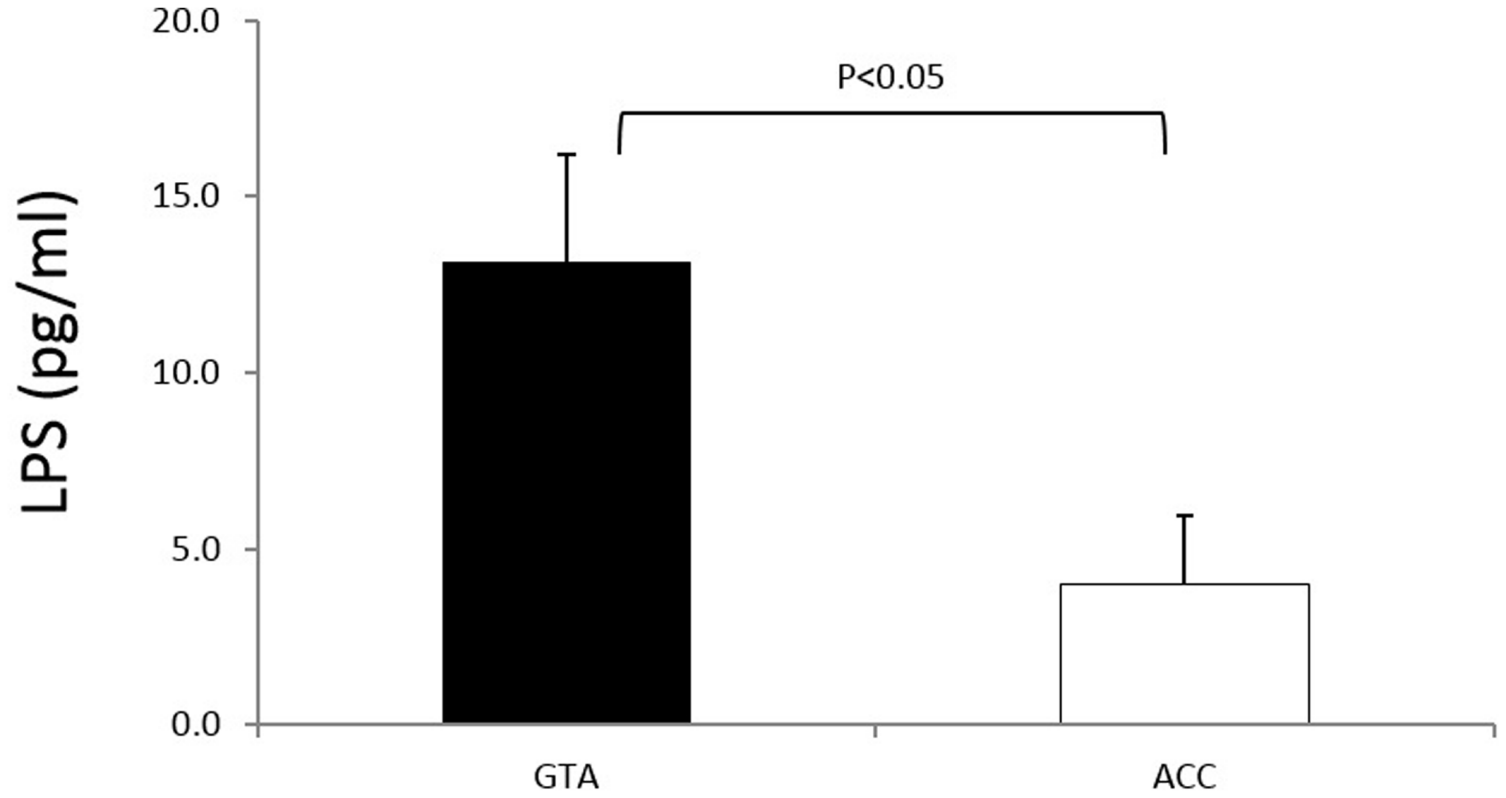
Plasma LPS concentration in obese subjects carrying the VDR “at-risk” (GTA) (black bars) or the “protective” (ACC) (white bars) haplotype. Mean values ± standard error is shown.

Plasma LPS concentration is a marker of altered gut barrier permeability and has been reported to be associated with increased inflammation in obesity. Obese individuals carrying the “at-risk” VDR GTA genotype showed higher LPS plasma concentrations than obese subjects with the “protective” ACC haplotype (*p*< 0.05) suggesting a correlation between obesity and microbial translocation that could be responsible for the maintenance of the inflammatory state at systemic level (Figure 3).

### VDR gene expression

Finally, the possible effect of the GTA “risk” and the ACC “protective” VDR haplotypes on the expression of the VDR gene was analyzed. Results indicated the presence of a slight but consistent decrease in VDR expression was associated with GTA “risk” (0.917 (95%) IC: 0.416-2.054) as compared to the ACC “protective” (1.265 (95%) IC:0. 587-2.239) haplotype (Figure S1).

## Discussion

In the attempt to shed light on the complex relationship between the vitamin D/VDR axis, metabolic diseases and inflammation we performed genetic and immunologic analyses on a group of individuals enrolled in the RIYADH COHORT, a longitudinal epidemiologic study that is ongoing in Riyadh, the capital city of the Kingdom of Saudi Arabia. Results of these analyses showed that VDR polymorphisms located in 3′ haplotype block region of VDR gene correlate with obesity development. Furthermore, VDR haplotypes that are associated with statistically increased or reduced risks of obesity and with higher/lower BMI scores could be identified. Notably, genes that are part of the inflammosome complex, and cytokines that are the end product of the activation of such complex, were significantly up regulated in cells carrying the VDR haplotype that correlates with an increased risk of obesity and higher BMI scores; associated with such “at risk” VDR haplotype was also an increased plasma concentration of LPS and a reduced expression of VDR.

Analysis of possible associations between VDR polymorphisms and obesity showed the presence of a strong association of the rs731236 (G) and rs1544410 (T) VDR minor alleles with higher BMI values independently of age and sex. In particular haplotype segregation analyses revealed the presence of a significantly higher risk of obesity and increased BMI in individuals carrying the GTA (rs731236 (G), rs1544410 (T), rs7975232 (A)) haplotype, whereas the complementary ACC (rs731236 (A), rs1544410(C), rs7975232(C)) haplotype was associated to a reduced risk of obesity and lower BMI scores, possibly suggesting a protective role. Once “risk” and “protective VDR haplotypes were identified we focused the subsequent part of the analyses on examining whether these haplotypes would have been associated with a different degree of inflammation. Results indicated that the VDR GTA “risk” haplotype is indeed correlated with a more robust up regulation of a number of key proteins that take part in the inflammosome activity, resulting in a significantly increased production of proinflmammatory cytokines; this same “risk” genotype was also correlated with a lower expression of VDR mRNA. These results suggest that two different genetic types of obesity that could be differentiated based on VDR haplotypes might exist: a more severe form that is associated with higher BMI scores and a more relevant degree of inflammation, and a less important one that may not correlate with an important inflammatory status. Notably, a subgroup of obese individuals has been described who does not display the typical metabolic disorders associated with obesity. These individuals are defined as metabolic healthy obese (MHO) do not have insulin resistance, lipid disorders, or hypertension and are hypothesized to have lower risk of obesity-related complications [18], [19]. It will be interesting to verify whether the GTA “protective” haplotype is predominant in these individuals.

It has been convincingly shown that the vitamin D/VDR axis modulates the activity of the immune system, and that in turn the immune system plays a pivotal role in vitamin D metabolism [20], [21]. Different reports suggest that chronic inflammation is a key marker of obesity, the origin of inflammation during obesity and the underlying molecular mechanisms that explain its occurrence are nevertheless not fully understood. The importance of inflammation in obesity was analyzed in the animal model. Thus, recent data show that NLRP3-/- knockout mice do not increase weight when fed a high fat diet, and that elimination of NLRP3 expression prevents obesity-induced caspase-1 cleavage as well as IL1β and IL18 activation [22]. These results suggest a direct involvement of NLRP3-inflammosome in obesity, and are reinforced by results herein indicating that the VDR GTA “risk” haplotype correlates with a higher inflammatory response to LPS. mRNA expression for a number of genes, including those of the inflammosome component downstream signaling and effector molecules was indeed significantly augmented in GTA obese individuals. These results were further confirmed by quantification of proinflammatory cytokines (IL1β, IL18, TNFα and IL-6) in LPS-stimulated cell cultures, suggesting that the degree of inflammation is directly correlated to obesity.

Plasmatic LPS concentration, a marker of altered gut barrier permeability, was also increased in GTA obese individuals. LPS binds to TLR4, a molecule expressed on the surface of a number of different immune cells. Recent results indicate that obesity-associated inflammation, besides being driven by dietary factors and nutrients such as glucose and lipids, is mediated by LPS [23], [24]. Indeed, fat deposition compromises intestinal permeability, liver function and the ability of Kuppfer cells to adsorb endotoxin [25], [26], whose circulating concentration is, therefore, increased. This in turn, mediates chronic low-grade of inflammation through the activation of TLR and the inflammosome pathway to exacerbate the insulin resistant state [27]–[30]. In agreement with these observation LPS plasma concentration was increased in obese individuals carrying the “at-risk” GTA genotype, suggesting a correlation between obesity and microbial translocation that could be responsible for the maintenance of the inflammatory state at systemic level. Increased systemic LPS concentrations are seen in HIV-infected individuals and are possibly responsible for the chronic inflammation that characterizes this condition [31]–[33].

Results obtained in *in vitro* systems indicate that the release of proinflammatory cytokines by human PBMC and rat peritoneal mesothelial cells is reduced by VDR agonists [10]–[12]. Recent data also indicate that the siRNA-induced down regulation of VDR expression results in an increased production of such cytokines by human adipocytes [13]. In this light it is interesting to note that a slight but consistent reduction of VDR expression was detected in cells that were characterized by the presence of the GTA “risk” haplotype. Thus the inflammation seen in the GTA haplotype could be the result of a different modulation of VDR expression, reinforcing the hypothesis of an additive effect of environmental and genetic factors in determining BMI [34].

In conclusion, results herein reinforce the idea that the vitamin D/VDR axis plays a role in the pathogenesis of obesity that is dependent on the presence of particular SNP and is at least in part mediated by an ongoing degree of inflammation, possibly secondarily to microbial translocation. Understanding the mechanism underlying the inflammation that is associated with obesity could contribute to the identification of novel therapeutic strategies to prevent or treat this metabolic condition.

## Materials and Methods

### Patients and controls

Eight hundred-ninety one Saudi individuals were enrolled in the study; 402 of these subjects (45%) (239 males, 163 females; mean age: 42.7±10.4) were frankly obese (BMI ≥30kg/m^2^) and 489 (263 males, 226 females; mean age 36.4±15.2) were non-obese. A group of 87 subjects (47 males and 37 females; mean age 34.87±13.1) were classified as overweight (BMI  = 25-29.9 kg/m^2^) [17]. However this borderline group was too small to be considered as a separate group. These individuals are part of the Biomarker Screening in Riyadh Project (RIYADH COHORT), a capital-wide epidemiologic study taken from over ∼17,000 consenting Saudis coming from different Primary Health Care Centers (PHCCs). In brief, subjects were recruited from their homes using a random cluster sampling strategy. Consenting participants were then requested to visit the nearest participating PHCC for questionnaire administration, anthropometric measurement and blood extraction.

A questionnaire focusing on demographic information and past medical history was given to all subjects. Those with co-morbidities that needed medical attention or with medical complications (coronary artery disease, diabetes mellitus type 2, nephropathy, thyroid diseases and end stage renal or liver disease) were excluded from the study. Anthropometry included measurement of height (to the nearest 0.5 cm) and weight (to the nearest 0.1 kg); BMI was calculated as kg/m^2^. Written consent was obtained and ethics approval was granted by the Ethics Committee of the College of Science Research Center, King Saud University, Riyadh, Kingdom of Saudi Arabia (KSA).

### VDR SNP analysis

Genomic DNA of all the enrolled subjects was isolated from 200 µl of frozen whole blood collected in EDTA-containing tubes by using the Illustra blood genomic prep minispin kit (GE Healthcare Europe GMBH, Freiburg, Germany). VDR SNPs (rs731236, rs1544410, rs7975232) were evaluated by allelic discrimination Real-time PCR using pre-designed TaqMan probes (Applied Biosystems, Foster City, CA, USA). The PCR consisted of a hot start at 95°C for 10 minutes followed by 40 cycles of 94°C for 15 seconds and 60°C for 1 minute. Fluorescence detection takes place at a temperature of 60°C. All assays were performed in 10 µl reactions, using TaqMan Genotyping Master Mix on 96-well plates using an ABI 7000 instrument (Applied Biosystems). Control samples representing all possible genotypes and a negative control were included in each reaction.

### VDR gene expression

Total RNA was extracted from 1.5 ml fresh blood (collected from 56 healthy subjects unrelated with patients enrolled in the study) and processed within an hour after collection, and cDNA samples were prepared as previously described (18)VDR gene Q-PCR experiments were performed in 96-well plates using an ABI Prism 7000 Sequence Detection System (Applied Biosystems) and a pre-made TaqMan probe (assay ID: Hs_01045840_m1). Three housekeeping genes (GAPDH, ACTB, YWHAZ) were used for normalization (assay IDs: Hs_99999905_m1, Hs_99999903_m1 and Hs_03044281_g1 respectively). Amplification efficiencies of target and reference genes assessment was carried out before any calculation of expression using the REST software (http://gene-quantification.com). Serial dilutions in triplets of a pool of 20 cDNAs were used for each transcript (VDR, YWHAZ, GAPDH, ACTB). The software determines the slope with a logarithmic algorithm as well as an indication of the linearity of logarithmic alignment using Pearson's correlation coefficient. The efficiency (E) is in the range from 1 (minimum value) to 2 (theoretical maximum and optimum) and is calculated from the slope, according to the equation E = 10 ^[-1/slope]^. Real-time relative expression experiments were performed according to manufacturer's instructions. Briefly, 1 µl of cDNA was used in a final PCR reaction volume of 20 µl containing 10 µl of gene expression master mix, 8 µl of water and 2 µl of TaqMan probe. PCR cycles were as follows: 10 min at 95°C followed by 40 cycles of 15°C at 95°C and 1 min at 60°C. All reactions were performed in triplicate, with non-template control for each gene and one inter-run calibrator that removes the technical run-to-run variation between samples analyzed in different runs.

### Inflammosome-pathway analyses

One x 10^6^ peripheral blood mononuclear cells (PBMC) isolated from 28 obese (VDR haplotypes: 14 GTA and 14 ACC) and 28 non-obese volunteers (VDR haplotypes: 14 GTA and 14 ACC) were incubated for either 3 (mRNA expression) or 24 hours (protein expression) with medium alone or in the presence of 2 µg/ml LPS. RNA was extracted using the acid guanidium thiocyanate–phenol–chloroform method, dissolved in RNase-free water, and purified from genomic DNA with RNase-free DNase (New England Biolabs, Ipswich, USA). One microgram of RNA was reverse transcribed into first-strand cDNA in a 20 µl final volume containing 1 µM random hexanucleotide primers, 1 µM oligo dT and 200U Moloney murine leukemia virus reverse transcriptase (Clontech, Palo Alto, California, USA).

The inflammosome pathway was analysed in LPS-stimulated cells using a PCR array including a set of optimized real-time PCR primers on 96-well plates (SABiosciences Corporation, Frederick, MD, USA). This approach allows the monitoring of mRNA expression of 84 genes related to the inflammosome-pathway activation, plus five housekeeping genes, following the procedures suggested by the manufacturer. Controls are included in each array for genomic DNA contamination, RNA quality, and general PCR performance. Experiments were run on individuals pooled into 4 different groups each including 14 subjects: ACC obese, GTA obese, ACC non obese and GTA non obese. Results represent the mean value of the different targets analysed. The mRNA expression rate obtained in ACC non-obese individuals was assumed as baseline expression level

### Bead-based multiplex ELISA

Pro-inflammatory cytokine concentrations (TNFα, IL-6, IL-18, IL-1β) were assessed in supernatant of 2 µg/ml LPS-stimulated or unstimulated PBMC isolated from obese individuals carrying either the ACC or the GTA VDR haplotype (28/group) with the Bead-based Multiplex method for the Luminex Platform. Supernatants were obtained following 24-hours LPS stimulation. Cell cultures were centrifuged for 10 minutes at 300g and supernatants were collected and stored at -20°C until ELISA quantification.

### Plasma LPS Concentration

Plasma LPS concentration was measured in 40 obese subjects carrying either the “at-risk” (GTA)(N = 20) or the “protective” (ACC)(N = 20) VDR haplotype with the LAL Chromogenic Endpoint Assay (Hycult biotechnology, Uden, the Netherlands) according to manufacturer's instructions. LPS concentration was calculated in EU/ml and expressed in pg/mL (1EU/ml = 1pg/ml) and calculated based on a standard curve. Intra and inter-assay CV were 8.9% and 12.3% respectively.

### Statistical Analysis

Data were analyzed using the SPSS version 16.0 (SPSS Inc., Chicago, IL, USA). Biochemical parameters were expressed as mean ± standard deviation (SD). Independent Student T-test was used to compare these data in lean and obese subjects. Analysis of variance (ANOVA) was used to compare different genotypes in each SNP followed by Dunnett's Post Hoc test. Linear regression analysis and analysis of co-variance (ANCOVA) was used to compare the VDR genotypes adjusted for the co-variants age and sex. Significance was set at *p*<0.05. Haplotype frequencies were estimated by the Expectation-Maximization algorithm (EM algorithm) implemented in PROC Haplotype in SAS Genetics statistical software package (SAS institute, Cary, NC, USA). The standardized measure of linkage disequilibrium (LD), termed r^2^, was computed at pairs of SNP loci. Tests of departures from LD were performed by using the likelihood ratio test (LR test) of linkage disequilibrium as used in PROC Allele of SAS Genetics. The most common haplotype was used as the reference and rare haplotypes were dropped from the analysis.

## Supporting Information

Figure S1 Relative expression of the VDR gene in cells of obese subjects carrying the VDR at “risk” (GTA) or the “protective” (ACC) haplotype. The boxes stretch from the 25th to the 75th percentile; the lines across the boxes indicate the median values, those stretching from the boxes indicate extreme values.
(TIF)
